# Severe Hypokalemia After Corticosteroid Use: A Case of Steroid-Induced Potassium Depletion

**DOI:** 10.7759/cureus.101479

**Published:** 2026-01-13

**Authors:** Justin D Tse, Abiolah Telesford, Maryam Shams, Neeladri Misra

**Affiliations:** 1 Internal Medicine, Graduate Medical Education, Sutter Roseville Medical Center, Roseville, USA

**Keywords:** corticosteroid-induced hypokalemia, corticosteroids, hypokalemia, potassium-wasting syndromes, rta

## Abstract

Hypokalemia can cause muscle weakness and life-threatening arrhythmias. Glucocorticoids can lower potassium through mineralocorticoid receptor activity and enhanced distal sodium reabsorption, and extensive topical corticosteroid use can also contribute. Clinicians must be able to identify the source of potassium loss and correct the deficit quickly.

Here, we present a 35-year-old man with hypertension, obesity, and obstructive sleep apnea who developed five days of weakness soon after starting a prednisone 50 mg taper and triamcinolone 0.1% cream. Serum potassium was 1.9 mmol/L with creatinine 0.94 mg/dL. Spot urine analysis showed an osmolality of 551 mOsm/kg, potassium of 5 mmol/L, creatinine of 121 mg/dL, and chloride of 60 mEq/L. The urine potassium-to-creatinine ratio was 4.1 mEq/g, which is below the threshold that indicates renal potassium wasting in hypokalemia. The transtubular potassium gradient was 1. There was no ECG abnormality. He received 320 mEq of potassium with improvement to 5.1 mmol/L. After stopping steroids and acyclovir, potassium normalized with no sequela after six weeks.

Severe hypokalemia can be multifactorial in new steroid users. In this patient, a significant total body potassium deficit, accompanied by a low transtubular potassium gradient, suggested non-renal losses with superimposed steroid exposure and other contributing factors. This case is notable for severe hypokalemia with low urinary potassium indices shortly after starting oral prednisone and large‑area topical triamcinolone, with normalization after withdrawal and repletion. When initiating systemic or high surface area topical corticosteroids, clinicians should monitor for side effects, including hypokalemia, review concurrent agents that affect tubular function, and use urine indices to localize losses. Early recognition of potential triggers can prevent arrhythmia and reduce complications.

## Introduction

Potassium plays a crucial role in maintaining cellular membrane potential, facilitating muscle contraction, and enabling the transmission of nerve impulses. Hypokalemia is a prevalent electrolyte disturbance defined by a serum potassium concentration of less than 3.5 mmol/L. Medications, especially diuretics and glucocorticoids, account for a substantial share of cases. The etiology of hypokalemia is varied, encompassing factors such as reduced dietary intake, increased cellular uptake, excessive renal or gastrointestinal losses, and medication-induced effects, most commonly from diuretics but also from agents like corticosteroids. Diuretics are the most common cause of drug-induced diuresis, but corticosteroids also increase distal sodium reabsorption and can promote kaliuresis through activation of the epithelial sodium channel. Severe hypokalemia can result in profound clinical manifestations, including muscle weakness, cardiac arrhythmias, and metabolic disturbances, highlighting the importance of timely recognition and effective management [1-4]. Severe hypokalemia can be multifactorial in new corticosteroid users. In this patient, urine indices, including a low transtubular potassium gradient and low urine potassium to creatinine ratio, suggested non-renal potassium loss with appropriate renal potassium conservation.

## Case presentation

A 35-year-old male with a past medical history significant for hypertension, class II obesity, and obstructive sleep apnea (OSA) presented to the emergency department with generalized weakness. He arrived at the Emergency Department reporting a five-day history of progressive fatigue and generalized weakness. He did not recall any specific triggers for his symptoms. The patient reported a recent worsening rash on the left side of his abdomen. He was initially seen at an urgent care center, where he was prescribed a 50 mg prednisone taper and triamcinolone 0.1% cream for his rash, with limited symptom relief. His social history was otherwise unremarkable for any substance use, including alcohol, cigarette smoking, or recreational drugs. Other home medications included metoprolol, clonidine, hydralazine, irbesartan, pantoprazole, and trazodone.

On admission, the patient’s vital signs were within normal limits, with the exception of an elevated blood pressure of 149/92 mmHg. A physical exam was unremarkable, aside from obesity, generalized fatigue, and a healing rash on the left side of the abdomen that was non-tender to palpation. Laboratory studies revealed a white blood cell count of 15.4×10^9^/L and a hemoglobin level of 18.1 g/dL. The basic metabolic panel results included severe hypokalemia, with a potassium level of 1.9 mmol/L, bicarbonate of 24 mmol/L, chloride of 110 mmol/L, while his creatinine, thyroid-stimulating hormone (TSH), C-reactive protein (CRP), and magnesium levels were within normal limits.

Chest X-ray demonstrated no pulmonary consolidation. An ECG was performed, revealing a normal sinus rhythm with no abnormalities. Given the patient’s severe hypokalemia, further evaluation of renal handling of potassium was pursued. Urine analysis indicated a urine osmolality of 551 mOsm/kg, a urine potassium level of 5 mmol/L, a urine creatinine of 121 mg/dL, and a urine chloride of 60 mEq/L. Urine sodium was not obtained in this sample. Urine potassium was 5 mmol/L, and urine creatinine was 121 mg/dL, yielding a urinary potassium-to-creatinine ratio of approximately 4.1 mEq/g, which is below the 13 mEq/g threshold that suggests renal potassium wasting in hypokalemia. The trans-tubular potassium gradient was 1, indicating that renal potassium losses were not the primary etiology. Urine pH was a single bedside dipstick value of 6, which suggested away from renal tubular acidosis (5, 6). Table 1 tabulates all laboratory values acquired on admission along with reference ranges.

**Table 1:** Lab results at the time of admission. mmol/L: millimoles per liter, mg/dL: milligrams per deciliter, mEq//g: milliequivalent per gram, mOsm/kg: milliosmoles per kilogram, K/uL: thousands of cells per microliter, g/dL: grams per deciliter, μU/mL: micro units per milliliter, mg/L: milligrams per liter.

Relevant Labs	Value	Reference Range
Sodium (mmol/L)	140	135-145
Potassium (mmol/L)	1.9	3.6-5.1
Chloride (mmol/L)	110	96-110
Bicarbonate (mmol/L)	24	21-32
Anion gap (mmol/L)	6	2-12
Magnesium (mg/dL)	2.8	1.6-2.6
Blood urea nitrogen (BUN) (mg/dL)	15	6-22
Creatinine (mg/dL)	0.94	0.7-1.4
Glomerular filtration rate (GFR)	108	>60
Calcium (mg/dL)	9.4	8.5-10.5
Urine pH	6	5.5-7.5
Urine potassium (mmol/L)	5	12-62
Urine creatinine (mg/dL)	121	30-125
Urine chloride (mmol/L)	60	55-125
Urine osmolality (mOsm/kg)	551	40-1400
Serum osmolality (mOsm/kg)	288	278-305
Trans tubular potassium gradient (TTKG)	1	<3
Urine potassium-to-creatine ratio (mEq/g)	4.1	<13
White blood cell count (K/uL)	15.4	4.5-11
Hemoglobin (g/dL)	18.1	13.5-17.5
Thyroid-stimulating hormone (μU/mL)	1.2	0.4-4.2
C-reactive protein (mg/L)	1	<1

Further history revealed that the patient had self-administered two doses of 300 mg acyclovir, which he had obtained from his wife, in an attempt to address his fatigue, which he had attributed to a possible viral infection. The medication use was factored into his clinical assessment, though it was not deemed a primary causative factor for his hypokalemia.

The patient was initiated on a potassium repletion protocol, requiring a cumulative total of 320 mEq of potassium to restore his serum potassium to 5.1 mmol/L. His symptoms showed gradual improvement as his potassium normalized, with the resolution of his fatigue. He was subsequently discharged in stable condition, with follow-up arranged through his primary care physician. Symptoms progressed over five days from fatigue to proximal limb weakness, and potassium normalized with repletion; outpatient potassium remained normal on follow-up at six weeks, without recurrence.

## Discussion

This case shows the multifactorial nature of severe hypokalemia in a patient with common comorbidities. Hypokalemia poses significant clinical dangers if left untreated. While mild cases may manifest as muscle weakness and cramps, severe hypokalemia can lead to potentially life-threatening complications such as cardiac arrhythmias, contributing to higher mortality. Potassium is a crucial electrolyte involved in several physiological processes, including the maintenance of cellular membrane potential, muscle contraction, and nerve impulse transmission. Rapid or severe depletion of potassium disrupts these essential processes, necessitating urgent medical intervention. Hypokalemia affects approximately 20% of hospitalized patients and is associated with a two-fold increase in mortality risk [1,2]. Specifically, serum potassium levels below 2.5 mEq/L significantly increase the risk of adverse outcomes, underscoring the critical need for prompt management [1-8]. This case illustrates the multifactorial nature of severe hypokalemia in a patient with common comorbidities.

When evaluating causes of hypokalemia, it is important to identify contributing factors as well as whether the origins stem from primary renal dysfunction. In severe hypokalemia, pair a spot urine potassium‑to‑creatinine ratio with the trans tubular potassium gradient (TTKG) and acid-base status, and if uncertainty persists, repeat spot testing or obtain a 24‑hour urinary potassium to confirm nonrenal versus renal losses [2,9]. In hypokalemia, a TTKG less than 3 to 4 indicates appropriate renal conservation, whereas higher values suggest renal potassium wasting; the patient’s TTKG near 1 supports a nonrenal mechanism or reduced intake [9,10]. In this case, urine potassium was measured at 5 mmol/L, and the trans-tubular potassium gradient was 1, suggesting that the kidneys were appropriately retaining potassium in times of depletion and that renal potassium losses were not the primary etiology. Additionally, the normal urine potassium-to-creatinine ratio and normal urine pH effectively ruled out renal tubular acidosis and potassium-wasting syndromes such as Gitelman, Bartter, and Liddle syndrome. He was not on any thiazide diuretics, which could explain anti-hypertensive iatrogenic causes, and he had no relevant social history to explain his losses. Although minimal, the patient's presenting symptoms of nausea, vomiting, and diarrhea likely compounded his hypokalemia because of gastrointestinal losses. GI potassium loss alone usually requires significant fecal potassium losses, and many hypokalemic diarrhea patients have fecal potassium levels below that threshold; therefore, reduced intake and volume status often contribute together [11, 12, 13]. Gastrointestinal potassium loss was considered, given the normal anion gap, though the overall presentation suggested a multifactorial etiology. Additionally, OSA can activate the renin-angiotensin-aldosterone system; however, the low TTKG and low urine K/Cr ratios here argue against OSA as the primary driver [14,15,16,17]. However, to have a deficit requiring 320 mEq of potassium for repletion is abnormal, warranting further evaluation. The combination of a low urine K/Cr ratio, a TTKG near 1, and rapid correction with repletion supports nonrenal loss and intake deficit as leading contributors in this episode.

Although acyclovir has been associated with hypokalemia through tubular dysfunction, reported cases primarily involve intravenous administration and acute kidney injury. This mechanism could have further complicated the patient's presentation in this case. However, acyclovir‑associated hypokalemia is reported mainly with intravenous dosing and often with acute kidney injury, so with normal creatinine here and brief oral exposure, acyclovir was unlikely the dominant cause [18,19].

The recent initiation of a prednisone taper for his rash, however, raised concern for steroid-induced hypokalemia as a primary etiology. Glucocorticoids, such as prednisone, are well-documented for their role in enhancing renal potassium excretion, thereby contributing to significant electrolyte disturbances. It is known that glucocorticoids also have an affinity for mineralocorticoid receptors (MR) in renal tubular cells, which can mimic the actions of aldosterone. Activated MR receptors can lead to the upregulation of sodium transporters, such as the epithelial sodium channels (ENaC), on the luminal side of principal cells in the collecting duct. Concomitantly, glucocorticoids can also stimulate the sodium-potassium ATPase pumps on the basolateral membrane, enhancing sodium reabsorption and potassium excretion. Ultimately, glucocorticoids can increase renal potassium excretion through mineralocorticoid receptor activation and ENaC‑mediated sodium reabsorption, and they can also precipitate transcellular potassium shift [9,20-22]. In our case, urinary indices favored appropriate renal conservation, so any steroid effect likely reflected transcellular shift rather than sustained renal wasting [10,20]. Even in steroid-naïve individuals, corticosteroid use can induce hypokalemia through their mineralocorticoid-like activity, which promotes renal potassium wasting. Furthermore, topical corticosteroids, such as triamcinolone, despite their localized effects, can lead to systemic potassium loss when used extensively or on large surface areas. This phenomenon still warrants further investigation [21-24]. 

The potential impact of triamcinolone and other corticosteroids on potassium homeostasis is significant, especially when combined with other potassium-depleting agents, and has been previously documented in clinical literature [8]. However, current case reports document renal tubular toxicity with acute kidney injury related to acyclovir, but this was not present in our patient [10]. It can be speculated that there was an interplay between acyclovir and prednisone use in a steroid-naive individual. This subclinical renal impairment may have compromised the patient’s ability for potassium homeostasis, further compounded by the concomitant use of steroids. The combined effects of acyclovir and prednisone most likely synergistically contributed to potassium depletion, further compounded by the patient’s history of OSA and more acute gastrointestinal losses. Prednisone and topical steroid exposure likely acted as permissive factors via transcellular shift, with nonrenal loss and low intake as leading contributors, rather than a single steroid-mediated renal wasting mechanism [9,20-22]. After discontinuation of steroids and acyclovir, the patient’s potassium normalized, and he was discharged with no complicating sequela.

## Conclusions

This case underscores the critical importance of a comprehensive approach in diagnosing and managing severe hypokalemia, especially in patients with multifaceted presentations involving several potential contributing factors. Although this is a single patient report and generalizability is limited, the diagnostic steps and urine indices are directly applicable to similar presentations. Because sampling was limited to a single pre‑repletion timepoint and intake and stool volumes were not quantified, conclusions rely on pattern recognition rather than serial confirmation. The role of corticosteroids, such as prednisone and triamcinolone, in precipitating hypokalemia, particularly when compounded by gastrointestinal potassium losses, emphasizes the necessity for vigilant monitoring and timely therapeutic intervention. This case serves as a reminder of the significant adverse effects associated with corticosteroid use and highlights the impetus for healthcare providers to remain vigilant when managing patients with complex electrolyte disturbances.
